# Equity and the financial costs of informal caregiving in palliative care: a critical debate

**DOI:** 10.1186/s12904-020-00577-2

**Published:** 2020-05-19

**Authors:** Clare Gardiner, Jackie Robinson, Michael Connolly, Claire Hulme, Kristy Kang, Christine Rowland, Phil Larkin, David Meads, Tessa Morgan, Merryn Gott

**Affiliations:** 1 Health Sciences School, Division of Nursing & Midwifery, The University of Sheffield, Barber House Annexe, 3a Clarkehouse Road, Sheffield, S10 2LA UK; 2 School of Nursing, University of Auckland, Auckland, New Zealand; 3 School of Nursing, Midwifery and Health Systems, University College Dublin, Dublin, Ireland; 4 Institute of Health Research, University of Exeter Medical School, Exeter, UK; 5 School of Health Sciences, The University of Manchester, Manchester, UK; 6 Institute of Higher Education and Research in Healthcare – IUFRS, University of Lausanne, Lausanne University Hospital, Lausanne, Switzerland; 7 Academic Unit of Health Economics, University of Leeds, Leeds, UK; 8 Department of Public Health and Primary Care, University of Cambridge, Cambridge, UK

**Keywords:** Palliative care, End of life care, Financial, Economic, Costs, Family carer, Informal carer, Caregiver, Equity, Inequity

## Abstract

**Background:**

Informal caregivers represent the foundation of the palliative care workforce and are the main providers of end of life care. Financial pressures are among the most serious concerns for many carers and the financial burden of end of life caregiving can be substantial.

**Methods:**

The aim of this critical debate paper was to review and critique some of the key evidence on the financial costs of informal caregiving and describe how these costs represent an equity issue in palliative care.

**Results:**

The financial costs of informal caregiving at the end of life can be significant and include carer time costs, out of pocket costs and employment related costs. Financial burden is associated with a range of negative outcomes for both patient and carer. Evidence suggests that the financial costs of caring are not distributed equitably. Sources of inequity are reflective of those influencing access to specialist palliative care and include diagnosis (cancer vs non-cancer), socio-economic status, gender, cultural and ethnic identity, and employment status. Effects of intersectionality and the cumulative effect of multiple risk factors are also a consideration.

**Conclusions:**

Various groups of informal end of life carers are systematically disadvantaged financially. Addressing these, and other, determinants of end of life care is central to a public health approach to palliative care that fully recognises the value of carers. Further research exploring these areas of inequity in more depth and gaining a more detailed understanding of what influences financial burden is required to take the next steps towards meeting this aspiration. We will address the conclusions and recommendations we have made in this paper through the work of our recently established European Association of Palliative Care (EAPC) Taskforce on the financial costs of family caregiving.

## Background

The benefits of palliative care for those approaching the end of life and their families are universally acknowledged. Despite this there is sparse evidence on the costs, cost effectiveness and equity impact of palliative care, in part due to the difficulties associated with measuring costs and outcomes in this context [1, 2]. One of the key issues in measuring these costs is how to capture the economic value of informal caregivers (also called family caregivers). In this context, informal/family caregivers are those in a close supportive relationship with a patient, who share in the illness experience and undertake vital care work and emotional management. They are often, but not always, family members [3]. Informal caregivers represent the foundation of the palliative care workforce and are the main providers of end of life care [4]; it is estimated that they provide 75–90% of home-based care for people who are near the end of life. Moreover, demand for informal care is rising as rapidly ageing populations mean that people are living longer with more complex health and social palliative care needs [5]. In this context palliative care refers to the care of those with life threatening illness, although it is acknowledged that many of the issues identified here will also apply to carers of those with chronic conditions.

Many countries have in recent years adopted palliative care policy which shifts the focus of palliative care provision out of hospitals and into the community [6]. An important but often neglected consequence of this policy shift is the impact on informal caregivers, for whom a move out of hospital and into the community would have significant implications [7, 8]. For example, a Canadian study in 2015 found that informal caregivers of patients dying at home spent more than twice as much time on unpaid caregiving compared with those caring for patients dying in hospital [9].

Financial pressures are among the most pressing concerns for many carers. In a recent Eurobarometer survey of preferences for government support, informal caregivers in Europe identified financial remuneration as their most important support need [10]. Whilst there is increasing recognition within government policies of the contribution of family carers, financial support can be difficult to access, dependent on complex eligibility criteria and often comes with a trade off in terms of restrictions on employment, further limiting opportunities for managing financial burden [4].

Furthermore, there is evidence that the costs of informal caring represent the latest in a growing list of equity concerns relating to palliative care. A 2015 report commissioned by Marie Curie detailed substantial inequities in access to specialist palliative care across the UK and similar evidence exists from other countries [11, 12]. Specialist palliative care is skewed towards those with cancer; is often poorly delivered in care-home settings; and is less available for older people, for those living in areas of social deprivation, for people from ethnic minority backgrounds, and for those with enduring mental illness [13, 14]. These are notable areas of concern, and we argue that the financial costs of caregiving should be recognised alongside other well recognised inequities in palliative care, and as a key social determinant of end of life experience. Such recognition is important if measures to address the financial impact of caregiving are to successfully address the current inequities outlined below. As Giesbrecht et al. (2012) argue: “without considering diversity, patterns in vulnerability and inequity are overlooked, and thus continually reinforced in health policy.” [15]

It is within this context that the European Association of Palliative Care (EAPC) has recently established a taskforce, with a view to furthering research and debate on the financial costs of informal caregiving in palliative care. The taskforce sits within the EAPC Reference Group for Family Carer research and comprises an international network of researchers, clinicians and policymakers. During a workshop held in Sheffield, UK over three days in July 2019, this interdisciplinary group critically reviewed the existing evidence, identified gaps in the evidence base, and proposed a strategy for further research. The most prominent issue identified was inequity in financial burden, therefore the aim of this critical debate paper is to summarise and critically discuss the evidence on this issue. First, we review some of the key evidence on the financial costs of informal caring, then we critically discuss a diverse evidence base which points to equity issues, and finish with recommendations for further research and how these may be achieved.

## Discussion

### What do we know about the costs of caring and the impact of these costs? A summary of existing evidence

Here we summarise a cross-section of the existing evidence in this area. This is not intended as a comprehensive or systematic review of evidence, rather a summary of recent key papers published in this area. Whilst we have attempted to include a range of international evidence, the lack of translation facilities means that literature from the UK and other English speaking countries predominates.

The financial costs of informal caring for a person approaching the end of life can be significant, and a small but expanding evidence base reflects the range and scope of these costs. A 2014 systematic review of literature on financial costs incurred by informal caregivers identified a very limited evidence base [16]. Nonetheless there was evidence to suggest that these costs are significant. Costs can be broadly categorised into three main areas: work related costs (costs related to changes in employment), carer time costs (cost related to time investment required by carers) and out-of-pocket costs (direct outlays of money). A 2015 qualitative study of bereaved carers confirmed that the costs of caring at the end of life are significant and include a range of both direct (e.g. transport, food, medication) and indirect costs (e.g. related to employment, carer time, carer health) [7]. The palliative care context was also found to increase costs, as meeting the ill person’s needs was prioritised over cost. Over recent years a growing number of international studies have confirmed that the financial costs of caring are a serious issue across the developed world [17–21]. Furthermore, evidence suggests that informal carers make a huge financial contribution to the wider healthcare system, with studies estimating that informal caregiving accounts for up to 70% of total health care costs [22].

Severe caregiving burden is experienced by many families as a result of financial problems and a lack of financial support. Major life changes are often required due to the cost of illness and caring, including moving house, delaying education or delaying medical care for other family members [7, 16]. Financial burden may also impact on employment, as carers may be forced to change their hours of employment to cope with increased financial expenditure, or to rely on annual leave or sick leave to maintain a salary [4]. The long term effects of these employment changes on job opportunities and earning potential are also cause for concern.

As noted above, the palliative care context can increase the financial burden for informal caregivers, and caregiver costs may increase as death approaches. In a study exploring the trajectory of palliative care costs over the last five months of life in Canada, informal care costs were found to increase significantly from the fifth month to the third month preceding death [17]. Similarly, Chai et al. (2014) [22] found that monthly unpaid caregiving costs increased exponentially with proximity to death. This evidence emphasises the distinct challenges faced by informal caregivers in palliative care. Next, we go on to discuss how these considerations influence the costs of caring and why some carers are more impacted than others, highlighting equity concerns related to financial support for informal carers.

### What is equity in healthcare, and why do the costs of informal caring represent an equity issue? A critical discussion

The evidence presented thus far outlines the financial impact of informal caregiving. Next, we consider some definitions relating to the concept of equity in healthcare. The distinction between equity and equality is important as equity, unlike equality, is a normative concept. Inequities in the use of health care are inequalities (differences) which are considered to be unfair or unjust [23]. Therefore, inequities only arise when variations in use between groups cannot be attributed to variations in need [24].

It is well established that people dying from cancer receive better access to specialist palliative care than those with non-cancer conditions [25], and this diagnosis related inequity extends to the costs of caring. A 2014 population-based study explored burden among informal carers of people at the end of life across four European countries [26]. The authors reported that in Belgium and Italy, carers of people with a non-cancer illness had significantly higher odds of having difficulties in covering costs, than carers of people with cancer. While this study provides some evidence on inequity related to diagnosis, the majority of research on the costs of caring focuses on carers of people with cancer, so the evidence base for non-cancer financial burden is sparse.

An additional challenge is that non-cancer carers may be ineligible for some types of financial support or benefits, which can exacerbate burden [27]. Across most developed countries charitable grant funding can be sought to help supplement a patient or carer’s income, in cases of extreme financial hardship. Grants are provided by a range of charitable organisations, however charities for those affected by cancer are the most prevalent and offer the most generous funding [27].

Another area of inequity is socio-economic status; those who are from lower socio-economic groups consistently face the most severe financial burden [28]. This in itself relates to education, as there is a strong correlation between level of education and wealth; across Europe those with a high level of education earn up to 70% more than those with a low level of education [29]. The consequences of financial burden for those who are already living in deprivation can also be catastrophic. Studies have reported that for those with limited financial resources, the financial costs of caregiving can result in having to move home, go without food, incur considerable debt [7] or resort to food banks and charitable handouts to meet even the most basic needs [27]. Finally, some people may have a choice about whether to care, but those who are less wealthy with limited financial reserves have less choice, as the alternatives are greatly limited [4]. Care work also relates to social class, and increasingly to migration. While some European countries still rely on local and/or family labour to provide paid and unpaid care, Western European countries increasingly rely on migrant labour to supply care. Women are also more likely to be both unpaid and home carers due to cultural norms and values. This impacts negatively on career and employment choices, often with long-term financial and health costs, such as loss of income, pension rights and failure to address their own health needs because of the burden of caregiving. Hence caregiving also raises questions of inequities related to gender, migration and culture [30].

There is considerable evidence globally to confirm that women are more likely to be caregivers for ill and ageing family members than men [31, 32]. Indeed, women report greater financial problems as a result of caregiving than men [33], although data specific to palliative care are limited and contradictory. As women are more likely to be caregivers than men, their opportunity costs are higher, particularly if viewed in terms of lifetime earnings. Many women take on sequential caring responsibilities up until advanced age, impacting upon the nature of employment they are able to pursue and their career advancement [34, 35]. Caring responsibilities also limit educational opportunities; this is particularly the case where there is a cultural imperative for young women to care for older family members [7]. Given the heavily gendered nature of caregiving in palliative care, we would argue that any research examining financial costs must consider both out of pocket and opportunity costs related to this work within a gendered context.

A number of studies have explored the impact of ethnicity and cultural identity on the financial burden of caregiving. With regards to ethnicity, the majority of studies report that ethnic minority groups are more vulnerable to financial hardship than white ethnic groups [36, 37]. Cultural identity can also influence costs. For example, in a qualitative study of bereaved caregivers in New Zealand, Māori carers faced more severe financial burden than non-Māori due to cultural values. These included the cultural imperative to return to ancestral homes before death and/or post death (tangihanga) which incurred additional transport costs [7].

Evidence is also beginning to emerge that employment status may represent an equity issue. A 2008 analysis of the British Household Panel survey (BHPS) revealed that those with caring responsibilities earned significantly less than those with no caring responsibilities [38]. Evidence indicates that working carers may have to take unpaid leave to provide care, reduce their working hours, change to a lower paid more flexible job, or give up work altogether [4]. Once these changes in employment have been made, it can be difficult to resume a pre-caring role, and many struggle to return to work after a period of providing care. The impact of these employment changes on a carer’s financial situation can be significant [4, 38].

Finally, in line with the broader turn in public health, the effects of intersectionality should be considered. This approach holds that individual’s experiences are shaped “not by a single axis of social division (such as gender, race, class) … but by many axes that work together and influence each other”. Whilst we have described a number of individual factors that may predispose carers to increased financial burden, certain groups of carers will fall into multiple categories and are likely to be most at risk due to the cumulative effect of risk factors. Applying an intersectional perspective Giesbrecht and colleagues (2012) found that culture, gender, geography, life-stage and material resources overlapped to explain people’s varied up-take of the Canadian Compassionate Care Benefit (a federal benefit which reimburses a caregiver’s earnings, so they can provide palliative care) [15]. In this vein, future research needs to acknowledge the full range of factors that could financially impact caregivers and where applicable conduct comparative analyses of economic costs across and/or within groups of caregivers to sensitively examine variation [39].

### Challenges and recommendations

The financial costs of family caregiving are a significant issue in palliative care, yet until now these costs have not been framed as an equity concern. Conceptualizing informal caregiver financial burden as an equity issue helps identify how this inequity can be addressed and draws attention more widely to the social determinants of care at the end of life, a key consideration under a public health approach to palliative care [12, 40].

Equity concerns are a persistent issue in palliative care; a wide range of factors are known to determine access to palliative care, and various social determinants are known to impact on the end of life experience. People with non-cancer diagnoses, older people [41], ethnic minority and indigenous groups, gypsies and travellers, homeless and LGBT people [42] and those from lower socio-economic groups [40] are all known to have less access to palliative care. Although these inequities persist, there is an increasing acknowledgement of a need for solutions. Policy options to address inequity include resources published by NHS England to improve end of life care for gypsies, travellers, LGBT people and homeless, those held in prisons [43], and those with learning disabilities [44]. To reflect the challenging picture across Europe, the EAPC has set up taskforces to address inequity in a number of the domains mentioned here, notably people with non-cancer diagnoses, prisoners and the LGBT community. As we have outlined above, the financial costs of informal caring represent another key area of inequity in palliative care, and in recognition we have had a proposal accepted by the EAPC to establish a new taskforce to develop research in this area and address some of the challenges identified here (https://www.eapcnet.eu/eapc-groups/task-forces/costs-of-family-caregiving).

One persistent issue which exacerbates these challenges is the lack of a whole system or societal perspective for the way we evaluate cost-effectiveness in palliative care. Regulatory authorities generally recommend that economic evaluations take the perspective of the health and social care provider. For example, in its reference case the National Institute for Health and Care Excellence (NICE) in the UK recommends a perspective of ‘NHS and personal and social services’ [45]. This does not include patients’ costs, caregiver time contributions to care, or caregiver costs [46]. Thus, at present an intervention could be shown to be cost-effective from the health perspective but actually increase costs for the carer. Furthermore, since current evaluations focus on efficiency and not equity, cost-effective interventions could also worsen inequalities. Methods for handling equity are available in health economics but at present are rarely used [47]. Whilst there has been some debate regarding the health and social care perspective (and indeed the NICE guidance is currently under review) [48, 49], it is still not clear how or whether a societal perspective should be implemented. Financial transfers which would be needed between different sectors may not be possible and difficult questions are posed regarding trade-offs between health, economic effects and other social considerations. In addition it is not clear how a range of activities including informal caregiver time contributions and impacts on employment ought to be valued [49]. Nonetheless, guidance elsewhere (for example from the 2nd panel on cost-effectiveness in the US) does recommend an additional broader perspective and this could adopted more widely [50].

A further challenge comes from the lack of a robust evidence base in this area, and difficulties with undertaking research on this topic. Evidence is required not only for resource rich countries, but also for low and middle income countries where the responsibilities of informal carers may differ. Evidence is also required which tackles how to support carers who are facing financial burden. A public health approach, challenging assumptions regarding who shoulders the financial responsibility of caring for the dying, would be appropriate. Methodological challenges also need to be considered [51], these include the sensitivity of discussing palliative care and financial issues, and stigma around welfare and benefits. Finally, a lack of consensus around appropriate designs and an absence of specialist data collection tools [52] contribute to the barriers to research. A commitment to co-production, working alongside patients and carers to design acceptable, sensitive and robust research is essential if we are to overcome these challenges and expand the evidence base in this important area.

Despite sparse evidence and the presence of regulatory systems which overlook informal carer contributions, we are able to suggest some policy recommendations for addressing inequity. Provision of financial or monetary support for informal carers through benefits is a clear and effective mechanism for reducing financial burden, however evidence suggests that this support is under-utilised and inequitably distributed in palliative care [27]. Policy options to mitigate this could include relatively subtle changes to the way financial support is implemented. For example, ensuring processes and systems do not unfairly penalise particular groups of patients/carers, consulting widely and across all groups when planning and implementing welfare change, relying on need rather than prognosis as a means of assessing eligibility and recognising that different groups of informal carers may have different needs. Communities and social networks may also play a role in recognising and supporting the contribution of informal carers. For example, the Compassionate City Charter is a set of principles which cover the civic aspect of our lives, including how we can become engaged in activities in the workplace which promote compassion and support continued employment [53].

There is also a potential role for health and social care professionals in recognising financial burden and signposting to appropriate support. The Carer Support Needs Assessment Tool (CSNAT) is an evidence-based tool developed for use in palliative care, which helps health and social care professionals work with carers to facilitate tailored, person-centred support [54]. Financial issues and work are assessed as part of CSNAT, emphasising the importance of these issues. If financial issues are identified then support such as an information leaflet detailing support for informal carers in palliative care could be appropriate, for example this information leaflet detailing carer support in the UK (https://www.sheffield.ac.uk/health-sciences/our-research/nursing-themes/palliative/financial-support-family-caregivers).

### Limitations

As this is a critical debate paper we have identified and critically appraised relevant literature but did not undertake a comprehensive or systematic evidence review, therefore some papers containing useful data may have been omitted. However, we did draw on previous relevant systematic reviews where they are available and the paper was written as a collaboration between subject experts and expert methodologists to help ensure an appropriate balance between evidence review and debate. Whilst we have attempted to provide a global oversight of evidence, we acknowledge that much of the evidence presented is from English speaking countries as we had no resource for translation costs. The perspective of non-English speaking countries is therefore particularly important to establish in future research.

## Conclusion

This review and critical discussion of literature has outlined the range and scope of the financial costs of informal caregiving and has provided evidence that these costs represent an equity concern in palliative care. We have described how various groups of informal carers are systematically disadvantaged financially. Addressing these, and other, determinants of end of life care is central to a public health approach to palliative care that fully recognises the value of the caring work undertaken by families, friends and their wider communities at end of life. Further research, exploring these areas of inequity in more depth and gaining a more detailed understanding of what influences financial burden is urgently required. Through the work of our recently established EAPC Taskforce we hope to be able to address some of these recommendations, and we welcome new members with interest and expertise – including postgraduate research students and earlier career researchers - to join us in this international endeavour.

## Data Availability

Supporting data can be accessed by contacting the lead author.

## Abbreviations

CSNAT: Carer Support Needs Assessment Tool
EAPC: European Association of Palliative Care
LGBT: Lesbian, gay, bisexual and transsexual
NHS: National Health Service
NICE: National Institute of Care Excellence
UK: United Kingdom
